# Crucial Breakthrough of Functional Persistent Luminescence Materials for Biomedical and Information Technological Applications

**DOI:** 10.3389/fchem.2019.00387

**Published:** 2019-05-31

**Authors:** Huaxin Tan, Taoyu Wang, Yaru Shao, Cuiyun Yu, Lidan Hu

**Affiliations:** Hunan Province Cooperative Innovation Center for Molecular Target New Drug Study, Department of Biochemistry and Molecular Biology, University of South China, Hengyang, China

**Keywords:** persistent luminescence material, biomedical applications, information technological applications, biosensing, optical data recording, anti-counterfeiting, therapy, bioimaging

## Abstract

Persistent luminescence is a phenomenon in which luminescence is maintained for minutes to hours without an excitation source. Owing to their unique optical properties, various kinds of persistent luminescence materials (PLMs) have been developed and widely employed in numerous areas, such as bioimaging, phototherapy, data-storage, and security technologies. Due to the complete separation of two processes, —excitation and emission—, minimal tissue absorption, and negligible autofluorescence can be obtained during biomedical fluorescence imaging using PLMs. Rechargeable PLMs with super long afterglow life provide novel approaches for long-term phototherapy. Moreover, owing to the exclusion of external excitation and the optical rechargeable features, multicolor PLMs, which have higher decoding signal-to-noise ratios and high storage capability, exhibited an enormous application potential in information technology. Therefore, PLMs have significantly promoted the application of optics in the fields of multimodal bioimaging, theranostics, and information technology. In this review, we focus on the recently developed PLMs, including inorganic, organic and inorganic-organic hybrid PLMs to demonstrate their superior applications potential in biomedicine and information technology.

## Introduction

Persistent luminescence (PL) is an optical phenomenon, in which luminescence is maintained for an appreciable time after the termination of the excitation (Hölsä, 2009). Although the origins of the PL emission are still in debate, the research, and applications of persistent luminescence materials (PLMs) have rapidly grown since the PL emission was first observed from a mineral barite (Bologna stone) in the 17th century (Lastusaari et al., 2012).

Following the enhancement in intensity, stability, and duration of PL, inorganic PLMs covering various emission colors have been fully studied and commercially applied. Since the first SrAl_2_O_4_:Eu^2+^,Dy^3+^ green emission PLM was discovered by Matsuzawa et al. (1996), persistent luminescence phosphors (PLPs) have been rapidly developed in the last decade (Matsuzawa et al., 1996). Recently, several PLPs emitting in visual spectra have been commercialized as important night-vision materials for various innovations, owing to their sufficiently strong, and ultra-long (>10 h) PL excited by sunlight or room light (Matsuzawa et al., 1996; Yamamoto and Matsuzawa, 1997; Wang et al., 2003). In the last few decades, visible light emitting PLPs have been widely utilized in critical applications including interior decorations, displays, signals and even certain newly-emerged technologies of anti-counterfeiting, optical recording, or biochemistry (Hölsä, 2009; Pan et al., 2012).

For biomedical applications, the PLMs can be ideal alternatives for traditional fluorescent materials, especially in bioimaging, due to the following two reasons (Lécuyer et al., 2016; Liu et al., 2018a). First, the entire fluorescence signal emitted from the PLMs *in vivo* because the tissue autofluorescence is eliminated owing to the termination of the excitation. Second, the delayed luminescence of PLMs facilitates long-time *in vitro* and *in vivo* bioimaging. In addition, the excitation and emission spectra of PLMs can be tuned conveniently to satisfy diverse demands. A typical example is near-infrared (NIR) luminescence, which is the most widely used excitation or emission wavelength in living imaging to achieve penetrability in deeper tissues (Wang et al., 2017a). However, a major limitation is the biocompatible size of PLMs. In 2007, PLM was synthesized in nanoscale in a pioneering study, initiating the research on persistent luminescence nanoparticles (PLNPs) (le Masne de Chermont et al., 2007). In addition to the above advantages, it has been verified by subsequent research in biomedical theranostics, that PLNPs have excellent dispersibility, biocompatibility and modifiability (Wang et al., 2017a; Sun et al., 2018; Xia et al., 2018).

Meanwhile, the novel generation of organic carbon-based PLMs ranging from small molecules to polymers has attracted significant attention. Compared with inorganic PLMs, the production of organic PLMs is more facile and controllable with reduced costs (Dimitrakopoulos and Malenfant, 2002; Kabe et al., 2016). The functionalization of organic PLMs with organic groups and biological ligands is more achievable. Moreover, their second level lifetime and environmental dependent feature is more suitable for demanding applications including display (Kabe et al., 2016), anti-counterfeiting (Zhou et al., 2015), bioimaging (Mikael et al., 2015), and sensors (Yang and Yan, 2016b).

With deeper understanding of the PL emission mechanism and the rapid development of synthesis technologies, more advanced applications based on PLMs have been explored. In this study, we review the crucial breakthroughs and the latest developments of research on PLMs with and without rare-element doping, to demonstrate their superior applications in biomedicine and information technology.

## Classifications

### Inorganic PLMs

#### Phosphors

Since the first SrAl_2_O_4_:Eu^2+^,Dy^3+^ PLM of green emission was discovered by Matsuzawa et al. (1996), PLPs have been rapidly developed in the last decade (Matsuzawa et al., 1996). Currently, several PL phosphors emitted in the visual spectrum, such as CaAl_2_O_4_:Eu^2+^, Nd^3+^ (blue emission), (Yamamoto and Matsuzawa, 1997) SrAl_2_O_4_:Eu^2+^,Dy^3+^ (green emission), (Matsuzawa et al., 1996), and Y_2_O_2_S:Eu^3+^,Mg^2+^,Ti^2+^ (red emission) (Wang et al., 2003), have been widely commercialized as important night-vision materials. Recently, researches have mainly focused on extending the emission and excitation spectrum and prolonging the PL duration.

**Figure 1:**
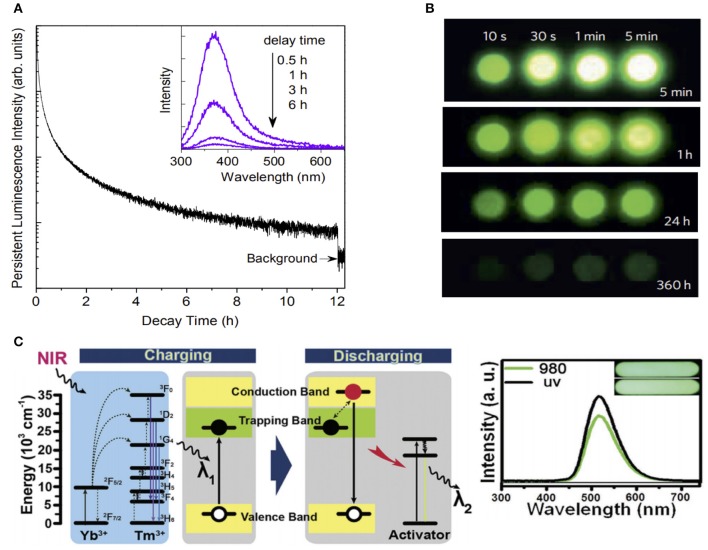
**(A)** PL decay curve monitored at 370 nm after irradiation by 254-nm UV for 15 min (Liang et al., 2015). **(B)** NIR images of four Zn_3_Ga_2_Ge_2_O10: 0.5%Cr^3+^ phosphor discs taken at different afterglow times after irradiation by a 365-nm lamp for durations ranging from 10 s to 5 min (Pan et al., 2012). **(C)** Energy transfer mechanism of UCPL materials (left), and the PL spectra of green emission UCPL materials taken 30 s after the excitation under 980-nm and UV lights for 15 s (right) (Hu et al., 2017). Reproduced with permission from Dalton Transactions, Nature Materials and Advanced Optical Materials.

To achieve an unchanged white afterglow color, an effective strategy is to combine different color emission from an identical luminescence center (Liu et al., 2005; Kuang et al., 2006). For example, by doping Tb^3+^ into Y_3_ Al_2_ Ga_3_ O_12_ host, three kinds of cross-relaxation energy transfer proceed: ^5^ D_3_ → ^5^ D_4_ and ^7^F_6_ → ^7^F_0_, ^7^D_J_ → ^5^D_1, 2, 3_ and ^7^ F_6_ → ^5^D_4_, and ^5^ D_4_ → ^7^F_4_ and ^5^ D_4_ → ^7^F_3_ in Tb^3+^, which are blue, green and red emissions respectively. By tuning the doping concentration of Tb^3+^, an unchanged white PL was obtained and maintained for more than 2 h (Zhang et al., 2017). In 2015, Pan and co-workers extended the PL into the ultraviolet (UV) spectral region. Using Pb^2+^ as luminescence center, the obtained Sr_2_MgGe_2_ O_7_:Pb^2+^ phosphors exhibited strong PL emission at 370 nm for more than 12 h (Figure 1A) (Liang et al., 2015).

Due to the high penetration depth and low autofluorescence, NIR-emission PLMs have attracted significant interest in biomedicine applications. For the NIR emission, Cr^3+^ is the main luminescent center with a narrow-band emission (700 nm) due to the spin-forbidden ^2^E→ ^4^A_2_ transition, and a broadband emission (650–1,000 nm) due to the spin-allowed ^4^T_2_→ ^4^A_2_ transition (Struve and Huber, 1985; Forster, 1990). In 2012, Pan and co-workers developed the Zn_3_Ga_2_Ge_2_O_10_:Cr^3+^ phosphors for the first time, which achieved an ultra-long NIR PL duration of 360 h (Figure 1B) (Pan et al., 2012). This breakthrough established Cr^3+^-doped gallates, such as ZnGa_2_O_4_:Cr^3+^ phosphors (Li et al., 2015b), LiGa_5_O_8_:Cr^3+^ phosphors (Liu et al., 2013), and Ca_3_Ga_2_Ge_3_O_12_: Nd^3+^,Cr^3+^ phosphors (Lin et al., 2016) as the preferred materials to obtain NIR PLMs. Meanwhile, Cr^3+^-doped non-gallate NIR PLMs (Zn_2_-xAl_2_xSn_1_-xO_4_:Cr^3+^), emitting with a strong 650–750 nm PL for more than 120s, have also been developed (Zhang et al., 2018)

To overcome the short-wavelength excitation limitation in deep tissues application, NIR-recharged PLMs were developed by introducing the upconversion concept (Chen et al., 2018). We fabricated NIR-rechargeable upconverting PL (UCPL) phosphors by combining UV-rechargeable PLPs with typical UV/blue emission upconversion materials (NaYF4:25%Yb, 0.5%Tm) (Figure 1C). It should be noted, that multicolor emission can also be realized by using different emission PL components. Compared with the UV excitation, no noticeable difference was found on the persistent phosphorescence properties under the NIR (980 nm) excitation (Hu et al., 2017, 2018).

#### Nanoparticles

While PLPs have been synthesized and maturely used for more than 20 years, their advanced development for biological application started in 2007, when Scherman et al. introduced nanoscale PLMs in their pioneering work (le Masne de Chermont et al., 2007). The conventional strategies for the preparation of PLPs typically involved high-temperature calcination for the formation of lattice defects and trap centers, which were crucial for the afterglow property of phosphors. The inevitable costs were the large irregular size and poor dispersibility of the synthesized PLPs, which limited their biological and medical applications. To overcome the solid-state barrier, Scherman and co-workers developed a sol-gel synthesis approach for the production of the first biocompatible PLNPs (Ca_0.2_Zn_0.9_Mg_0.9_Si_2_O_6_ doped with Eu^2+^, Dy^3+^, and Mn^2+^) and application for *in vivo* imaging. Moreover, those nanoparticles exhibited strong persistent NIR luminescence *in vivo* for more than 1 h after being excited by UV light before injection.

In recent years, motivated by this, an increasing number of synthetic routes have been reported and summarized (Li et al., 2016). In 2017, Wang et al. outlined established techniques for PLNP preparation and classified them into two groups: the top-down and bottom-up approaches (Wang et al., 2017a). In the top-down approach, the PL of PLPs is formed in solid or sol-gel state via high-temperature combustion. From large to small, or top to down, the PLPs with large size are processed into PLNPs by certain physical methods such as grinding and pulsed laser ablation.

To achieve size- and shape-controlled building-up processes, the bottom to top methods, including hydrothermal synthesis and the template method, introduced new approaches for PLNP construction. To obtain PLNPs with diverse morphology, the synthetic processes are highly controllable by adjusting the reaction conditions. In 2015, Han et al. used a hydrothermal method for PLNP production for the first time (Li et al., 2015a). An 8-nm nanoparticle ZnGa_2_O_4_Cr_0.004_ PLNP was synthesized in solution, which possessed renewable NIR PL and exhibited excellent monodispersity under different aqueous conditions. Yang et al. synthesized ZnGa_2_O_4_:Cr^3+^ PLNPs with specific kiwifruit-like structures using silica as templates (Lin et al., 2017). The designed morphology could be conveniently controlled by changing the size and thickness of the silica templates, which ensured the NIR PL performance during high-temperature calcination. In addition to the size and shape, the superficial geography of PLNPs, which is critical for biomedical applications, can be easily modified in the bottom-up approach. Yan et al. reported dual-modal PLNPs, functionalized with hyaluronic acid (HA) modified Gd_2_O_3_ via hydrothermal and biomineralization synthesis (Wang et al., 2017c). The possible application of these versatile nanoprobes can be in PL and magnetic resonance (MR) imaging, as ZnGa_2_O_4_:Cr_0.001_ provides the NIR PL, Gd_2_O_3_ enhances the MR signals, and the HA can target tumor cells *in vitro* and *in vivo*.

### Organic PLMs

Inorganic PLMs doped with rare-earth elements exhibit excellent optical performance with high durability and long emission. However, these systems have low dispersibility and biocompatibility, and require complex fabrication process, which limit its future applications. To overcome these limitations, several types of organic PLMs have been investigated.

**Figure 2:**
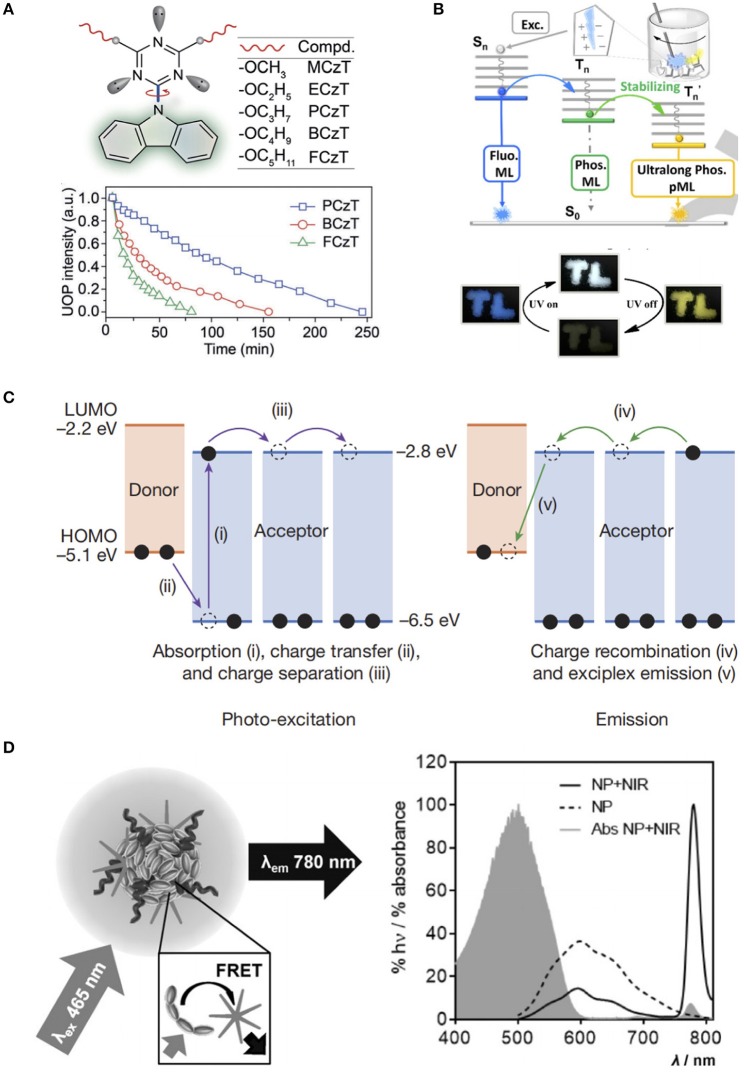
**(A)** Rational design of molecular rotors (top) and ultra-long organic phosphorescence deactivation process by monitoring the emission intensities at 530 nm under ambient conditions (bottom) (Gu et al., 2018). **(B)** Energy transfer processes for transient and persistent ML of ImBr (top) and tricolor emission switching of ImB (bottom) (Li et al., 2018). **(C)** Emission mechanism of organic PL (Kabe and Adachi, 2017). **(D)** Schematic of the FRET from the MEH-PPV polymer to the NIR775 dye (left), and fluorescence spectra of the NPs with and without NIR775 dye, and absorbance spectrum of the NPs with NIR775 (right) (Mikael et al., 2015). Reproduced with permission from Angewandte Chemie International Edition, Nature, and Angewandte Chemie, respectively.

Owing to the short-lived singlet exciton for fluorescence, it is a great challenge to achieve PL in purely small organic molecules, especially in single-component PL. In 2015, Huang et al. proposed that triplet excited states could be stabilized by strong coupling in H-aggregated structures. Based on this principle, a diverse array of purely organic-molecule PLMs was found with ultra-long second level luminescence lifetimes. By tailoring the molecule structures, the emission color can be tuned from green to red (An et al., 2015). In their subsequent research, a series of organic PLMs with a triazine core, carbazole unit, and alkoxy chains were designed (Figure 2A). The PL lifetimes were increased from ms to s by exploiting the rotor unit of carbazole and triazine, which could stabilize excited triplet excitons via the manipulation of intermolecular interactions (Gu et al., 2018). In 2018, Chi and co-workers presented a purely organic aggregation-induced emission (AIE) for the first time that exhibited transient, persistent photoluminescence, and persistent mechanoluminescence (ML) at room temperature (Li et al., 2018). As a key functional unit, N-(4-trifluoromethylphenyl)phthalimide was introduced, which was favorable to form crystals and to prevent non-radiative transitions by immobilizing the molecular conformations. Owing to its capability of promoting spin-forbidden transitions, a bromine substituent was used to enhance the intersystem crossing efficiency of the singlet-to-triplet excited state. The tricolor emission switching between blue, white, and yellow could be obtained by simply switching on and off the UV lamp (Figure 2B).

To achieve multi-component PL, a commonly used strategy is the host-guests system, which consists of phosphorescent guests and efficient non-radiative vibration- limiting hosts (Hirata et al., 2013). However, the practical preparation is rather complex owing to the requirement of the elaborate selection of host and guest molecules for good compatibility and the careful tuning of doping concentration to obtain the best PL performance. In 2017, Kabe and Adachi proposed a novel strategy to obtain multi-component PLMs, which only required a simple mixture of a strong electron donor N,N,N',N'-tetramethylbenzidine (TMB) with a strong electron acceptor 2,8-bis(diphenylphosphoryl)dibenzo[b,d]thiophene (PPT) (Kabe and Adachi, 2017). The gradual recombination of these two radical anions and cations provided a stable radical cation and high triplet energy, as well as a non-radiative suppressible rigid amorphous environment to generate exciplex emission (Figure 2C). More importantly, a breakthrough was achieved in the PL lifetime of the obtained PLMs, which was upgraded to the 1-h level. In their subsequent research, color-tuning emission from greenish-blue to red and even warm white was achieved by simply doping the organic PLMs matrix with a wide variety of emitter molecules. Due to the doped emitters, the exciplexes could generate energy via Förster resonance energy transfer (FRET), as well as prolong the emission by acting as electron trapping sites, which resulted in improved brightness and emission duration (Jinnai et al., 2018).

As important organic macromolecules, polymers were found to be suitable as PL emitters. In 2015, Rao et al. reported a certain type of fluorescent semiconducting poly[2-methoxy-5-(2-ethylhexyloxy)-1,4-phenylenevinylene] (MEH-PPV) nanoparticles, which generated NIR PL for 1-h after being excited by white light (Mikael et al., 2015). Owing to the long π-conjugation conducting bands of MEH-PPV, energy can be stored in the semiconducting layer. In the presence of NIR775, the released energy was transferred to the NIR dye encapsulated in the polymer nanoparticles and resulted in NIR PL (Figure 2D).

In addition to single component polymer PLMs, polymers of polylactic acid (PLA), poly-arylene ether phosphine oxide (PBPO), poly-methyl methacrylate (PMMA), poly-vinyl alcohol (PVA) and poly -vinyl-pyrrolidone (PVP) were also suitable as host materials to obtained multi-component PL by minimizing the non-radiative decay of the long-lived triplet excitons (Zhang et al., 2007; Al-Attar and Monkman, 2012; DeRosa et al., 2015; Gu et al., 2018). For example, a flexible organic PLMs system based on the engineering plastic PBPO was developed, and the PL of this system can be articles a for more than 7 min after low-power excitation. This polymer-based system exhibited excellent mechanical flexibility required for future applications such as curved products, fibers, and films (Yang and Yan, 2016b).

### Inorganic-Organic Hybrid PLMs

As kind of inorganic-organic hybrids, metal–organic frameworks (MOFs) possess rigid inorganic porous structure, which can capture and stabilize emitter molecules. Therefore, the coordination of organic fluorescent units with common metal ions is effective in achieving PL in MOFs. Recently, Yan and co-workers have devoted significant efforts to explore MOFs-based PLMs. By coordinating terephthalic acid and Zn^2+^, they developed Zn-isophthalic acid (Zn-IPA) MOFs for the first time, which exhibited green PL with a lifetime of 1.3 s (Yang and Yan, 2016b). To achieve color-tuning emission, N,N'-dimethylformamide (DMF) was introduced into the MOF nanochannels and formed a novel MOFs system [Zn(TPA)(DMF)], which exhibited tunable PL colors from green to red and cyan to yellow (Yang and Yan, 2016a). To increase the lifetime of PL emission, deuterated coronene was introduced into the zeolitic imidazolate framework (ZIF). After being encapsulated within this coronene@ZIF, the non-radiative deactivation of the emitter was proven to be significantly reduced and finally enabled a long lifetime of up to 22.4 s (Mieno et al., 2016).

In addition, metal coordination polymer (CP) materials with conveniently tunable photoactive units and photo-emissive properties were also designed for PLMs. Yan et al. developed a series of Cd-based CPs by coordinating Cd^2+^ with 1,3-benzenedicarboxylic acid (BDC), which exhibited a noticeable ultra-long afterglow of 0.70 s (Yang et al., 2016a). To achieve color-tunable PL in CPs, a lanthanide cations doping approach was developed (Yang and Yan, 2017; Yang et al., 2017). The doped Eu^3+^ and Tb^3+^, which have a sufficiently broad absorption band, can capture energy from CPs and show an obvious red and green PL with lifetimes of 10.54 and 57.66 ms, respectively.

## Applications

### Biomedicine

#### Bioimaging

**Figure 3:**
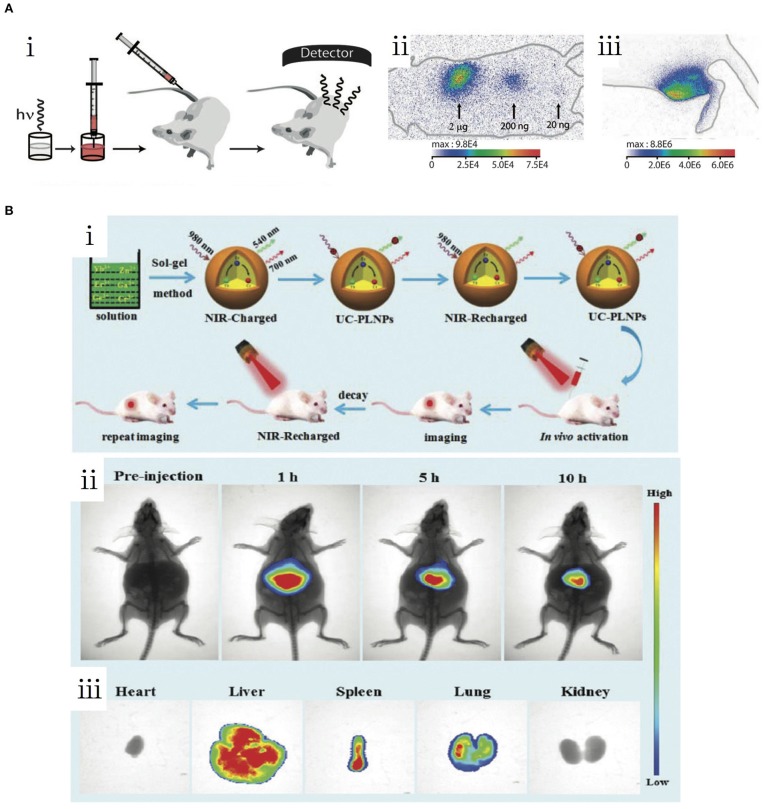
**(A)** First PLNP-based *in vivo* bioimaging (le Masne de Chermont et al., 2007). (i) Schematic of the experimental procedure of the *in vivo* imaging. (ii) Living image of NPs via subcutaneous injection. (iii) Living image of NPs via intramuscular injection. **(B)** NIR-to-NIR upconverted persistent probe for *in vivo* bioimaging (Zeng et al., 2017). (i) Schematic of the preparation and experimental procedure of UC-PLNPs for *in vivo* bioimaging. (ii) *In vivo* images of UC-PLNPs under 980-nm excitation. (iii) Biodistribution of UP-PLNPs in major organs. Reproduced with permission from copyright (2007) National Academy of Sciences and Nanoscale, respectively.

As mentioned in the previous section, PLNPs, especially those with NIR emission, are ideal probes in bioimaging for ultra-long PL without tissue autofluorescence background, compared with the conventional fluorescent probes such as quantum dots (Liu et al., 2018a). The first generation of PLNPs employed in *in vivo* imaging required pre-excitation by UV before being injected into the body (Figure 3A) (le Masne de Chermont et al., 2007). Instead of using short wavelength exciting light resources, the second generation of bioimaging PLNPs can be charged by visible lights, which enable their direct *in vivo* stimulation. Scherman et al. improved their results on living imaging by the synthesis of visible light stimulated ZnGa_1.995_Cr_0.005_O_4_ PLNPs (ZGO) (Thomas et al., 2014a). The ZGO exhibited long-last NIR luminescence excited by orange/red light-emission diode (LED). Following the injection of RAW 264.7 cells with intracellular ZGO, based on the PL signals activated by LED the cells could be traced real-time *in situ* for more than 24 h. To fully exploit the tissue transparency widows in the NIR region, hybrids designs of upconversion materials and PLNPs became widely used. In 2017, Zeng and coworkers reported a combination of upconversion and PL based on the construction of upconversion-PLNPs (Zn_3_Ga_2_GeO_8_:Yb/Er/Cr) (Figure 3Bi) (Zeng et al., 2017). Due to the upconversion effect, the nanocomposites can be excited by a 980-nm laser and emit 700 nm light, which can be absorbed and stored by PLNPs to produce NIR emission signals. Furthermore, studies demonstrated rechargeable *in vivo* PL for a duration of more than 10 h, which can achieve actual NIR-to-NIR bioimaging (Figures 3Bii,iii).

#### Therapy

**Figure 4:**
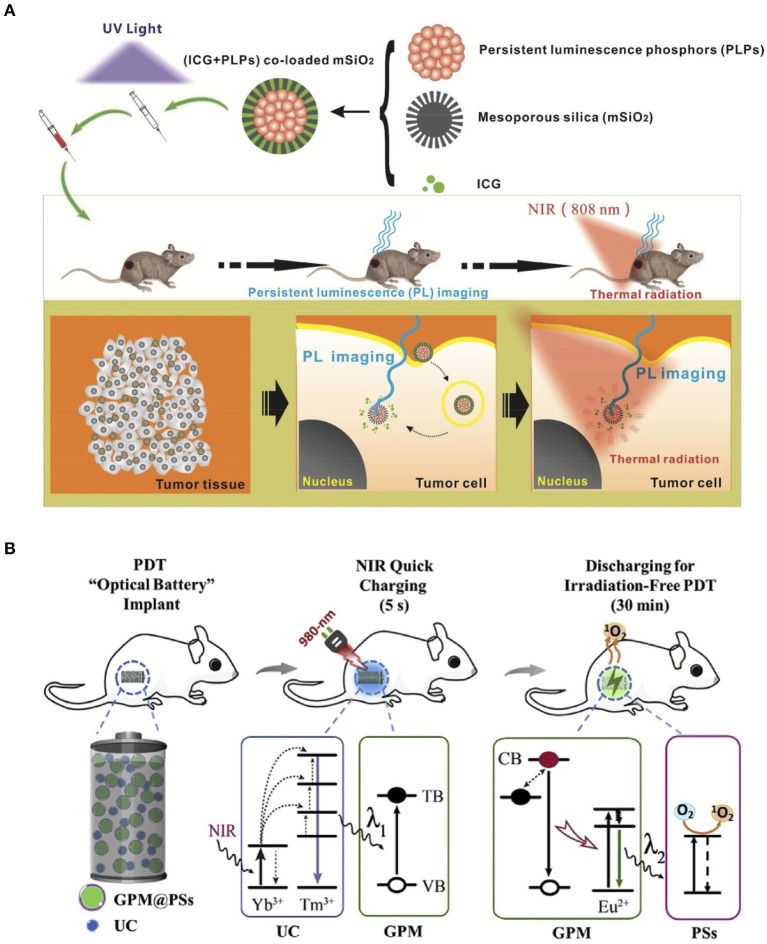
**(A)** Photothermal therapy using ICG-PLPs@mSiO_2_ (Zheng et al., 2016). **(B)** Photodynamic therapy using NIR light rechargeable UC-PLNPs (Hu et al., 2018). Reproduced with permission from ACS Applied Materials and Interfaces and Biomaterials, respectively.

By exploiting the PLMs irreplaceable optical nature, the PL-based theranostic applications typically combined with other therapeutic materials, can be implemented in imaging-guided chemotherapy, photothermal therapy (PTT), and photodynamic therapy (PDT) (Liu et al., 2018a; Sun et al., 2018). In 2014, Richard et al. proposed a hybrid nanostructure containing ZnGa_1.995_Cr_0.005_O_4_ PLNPs and mesoporous silica as an anticancer drug delivery system (Thomas et al., 2014b). The doxorubicin-loaded ZGO@SiO_2_ potently inhibited the growth of U87MG cells than the empty vectors *in vitro*. The *in vivo* transportation process was monitored in real-time. As the only FDA-approved water-soluble photothermal agent, indocyanine green (ICG) is often used in PTT systems. Chang et al. presented a novel design combining ICG and PLPs@mSiO_2_ (Zheng et al., 2016). Composite materials, exhibiting both PL and photothermal conversion properties, are suitable for imaging-guided PTT (Figure 4A). The PL-based PDTs use a combination of PLMs and photosensitizers, which can generate cytotoxic singlet oxygen to damage the targeted cells. Zhang et al. developed a PLMs-based irradiation-free photodynamic therapeutic method by integrating the advantages of PLMs, upconversion materials, and photosensitizer (Figure 4B) (Hu et al., 2018). The combination of NaYF_4_:25%Yb, 0.5%Tm upconversion materials and SrAl_2_O_4:_Eu^2+^,Dy^3+^ PLMs enabled green PL excited by NIR. By absorbing these persistent green lights, the attached rose bengal (RB) photosensitizers were able to continuously produce singlet oxygen. Owing to the high transparency of NIR and the renewability of inner PL, the implanted material, such as an optical battery, can implement effective PDT for *in vivo* tumor suppression.

#### Biosensing

**Figure 5:**
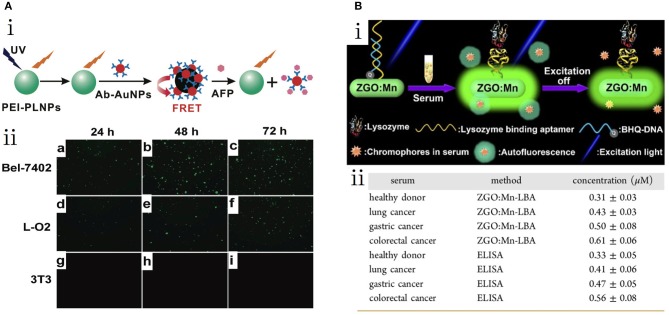
**(A)** PLNP-based detection of AFP (Wu et al., 2011). (i) Schematic of the AFP detection of PEI-PLNPs and Ab-AuNPs. (ii) Fluorescent images of three cell lines with PLNP-based probes. **(B)** PLNR-based detection of serum lysozyme (Wang et al., 2017b). (i) Schematic of lysozyme biosensing. (ii) Detected concentrations of lysozyme by PLNRs and ELISA. Reproduced with permission from Journal of the American Chemical Society and ACS Nano, respectively.

In 2011, Yan et al. introduced a PLNP system for biosensing (Wu et al., 2011). They combined PLNPs (Eu^2+^- and Dy^3+^-doped Ca_1.86_Mg_0.14_ZnSi_2_O_7_) and gold nanoparticles (Ab-AuNPs) modified by α-fetoprotein (AFP) antibody for the detection of AFP in tumor cells (Figure 5Ai). The AFP is a serum glycoprotein secreted by hepatic cells in newborn period only. The abnormal increase of AFP in the serum can strongly indicate several types of cancerization. The conjugation of PLNPs and Ab-AuNPs results in an FRET attributed to the overlap of PL emission and the AuNP absorption spectra. In this process, the excited energy is captured in the FRET system, which annihilates the emitted light of the nanocomposites. While introduced to AFPs, the competing antibody-antigen bonds released the PLNPs from the AuNPs, which produces long-lasting luminescence. By avoiding the *in situ* excitation, the PLNPs-based nanosystem exhibits high signal-to-noise ratio (SNR) and excellent sensitivity for the quantitative detection of AFP in serum samples and cancer cells with a minimum detectable concentration of 0.41 μg/L (Figure 5Aii). Yuan et al. developed a controllable hydrothermal synthesis method to obtain PL nanorods (PLNRs) functionalized for serum lysozyme analysis (Figure 5Bi) (Wang et al., 2017b). The further functionalization of PLNRs comprised two important parts: lysozyme-binding aptamers and a special DNA segment for luminescence quenching. The detachment of the DNA quencher together with the specific binding of lysozyme and the aptamer recovered the PL of PLNRs. The accuracy of lysozyme detection was comparable to that of ELISA in three clinical cancer samples with minimum a detectable concentration of 0.31 μM in a normal donor (Figure 5Bii).

### Information Technology

#### Anti-counterfeiting

With the rapid development of science and technology, innovative anti-counterfeiting technologies are highly pursued under the situation of increasing counterfeiting activities. In recent years, anti-counterfeiting technologies based on PLMs have received significant attention owing to their properties of high emission intensities and long recognizable time.

**Figure 6:**
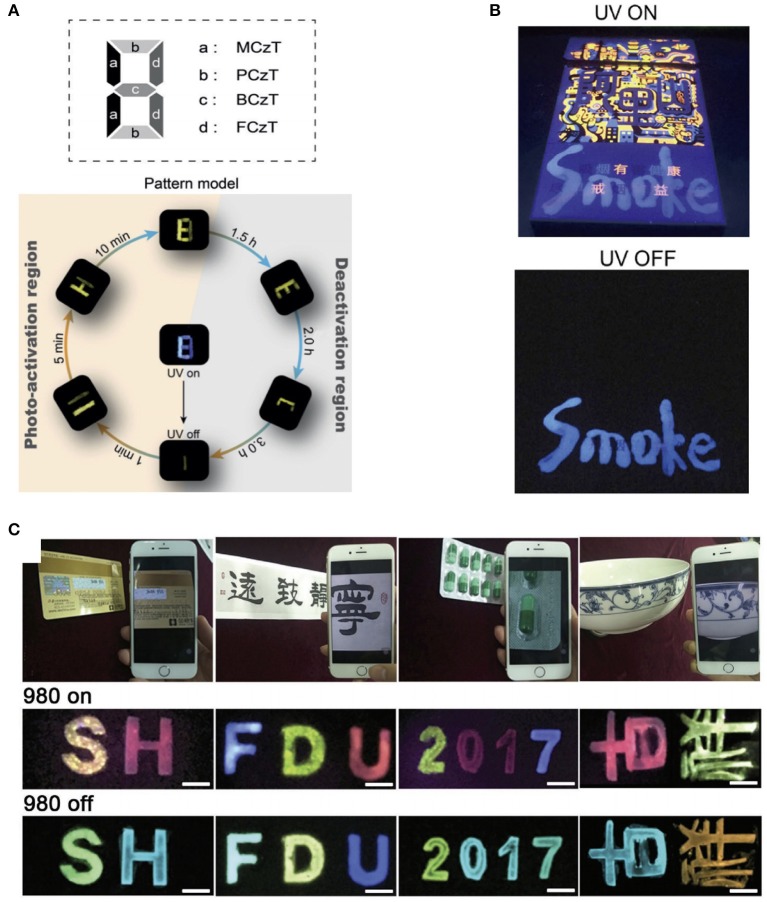
**(A)** Pattern designed with different phosphors and demonstration of multilevel anti-counterfeiting using MCzT, PCzT, BCzT, and FCzT crystals (Gu et al., 2018). **(B)** Photographs of handwritten characters on a cigarette case under a UV lamp (254 nm) and after the lamp is switched off (Gao et al., 2017). **(C)** Typical objects stamped with orthogonal multicolor UCPL materials and the luminescence images when the 980-nm excitation is switched on and off (Hu et al., 2017). Reproduced with permission from Angewandte Chemie International Edition, Nano Research and Advanced Optical Materials, respectively.

Their direct coverage capability on various substrates enables the application of the great potential of organic PLMs in anti-counterfeiting technologies (Deng et al., 2013; An et al., 2015; Jiang et al., 2016; Yang et al., 2016b; Xue et al., 2017). In 2018, Huang and co-workers prepared a series of organic PLMs with controllable tuning of PL photoactivation and deactivation times by customizing the alkoxy chains (Gu et al., 2018). The PL of these molecular compounds can be activated by prolonged photo irradiation, and can be deactivated by thermal treatment or UV irradiation for 3 h. Considering the unique dynamic PL features of these materials, the pattern “8,” which was built by different crystalline molecular compounds, could be converted into various digital numbers or letters of “8,” “11,” “I,” “H,” “E,” and “C” under different irradiation or stoppage irradiation conditions (Figure 6A). Gao et al. developed a layered double hydroxides based polymer (PMA/LDH@PAA) thin film with a tunable PL color for anti-counterfeiting applications (Gao et al., 2017). The transparent PMA/LDH@PAA was written on a cigarette case and the writing became invisible after thorough drying (Figure 6B). Upon UV irradiation, the bright blue word appeared. However, the PMA/LDH@PAA anti-counterfeiting mark was still apparent after the termination of UV irradiation, whereas the proprietary markers disappeared immediately.

Compared with organic PLMs, inorganic PLMs with longer PL time and higher emission intensity are more suitable to realize anti-counterfeiting recognizable with the naked eyes. In 2018, Liu et al. synthesized a series of NaBaScSi_2_O_7_-based phosphor with photoluminescence (PhL), PL, and photostimulated luminescence (PsL) properties using co-doped Eu^2+^/Nd^3+^/Pr^3+^ ions (Liu et al., 2018b). Under different light excitation conditions (the excited UV or 980-nm light was switched on or off), a difference in the emission colors of PhL, PL, and, PsL was represented by the different release processes of the carriers. Therefore, multiplex anti-counterfeiting was achieved by simply changing the doping of rare-earth ions in a single matrix. With the introduction of a novel NIR rechargeable orthogonal multicolor UCPL, an orthogonal anti-counterfeiting technique was developed by our research group (Hu et al., 2017). Using a simple method of stamp or modified inkjet printing, these composites enable high-speed patterned deposition on various mediums such as plastic, paper, or ceramics (Figure 6C). Each anti-counterfeiting letter formed has two sets of independent orthogonal emission colors recognizable with the naked eye: a multicolor upconversion luminescence when 980-nm NIR is switched on or a multicolor PL when it is switched off, which can be easily detected by a smartphone.

#### Optical Data Recording

**Figure 7:**
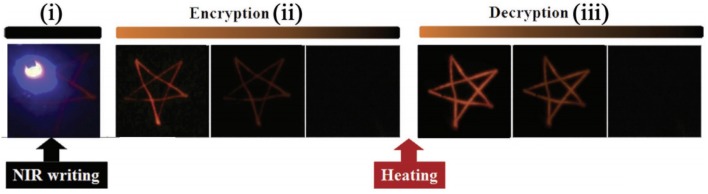
NIR-writing encryption process and heat-induced decryption process (Hu et al., 2017). Reproduced with permission from Advanced Optical Materials.

Due to the energy storing capability of the deep traps, a part of the excitation energy is stored for a long time after being exposed to the light. Then, this portion of the energy can be stimulated released by optical, thermal, or mechanical force, which results in emissions and long PL. Therefore, the special phenomenon of thermo-luminescence, photostimulated luminescence, and mechanoluminescence can be applied in information write-in and read-out processes. Our research group has constructed a data-recording device based on the NIR-rechargeable upconversion PLMs (Zhuang et al., 2018). This device was fabricated by simply dispersing PLMs into polydimethylsiloxane to form a film. A 980-nm laser was used as a “pen” to encode date on the device. Following the stopping of writing, this information faded gradually and disappeared completely. However, a portion of energy obtained from the pen was stored in deep electron traps and did not discharge under normal environment for a long time. Then, under heat treatment, the recorded information could be decrypted and appeared instantly with bright PL for a duration of 1 min, due to the thermo-luminescence properties (Figure 7). Light-luminescence properties were also used to developed data storage devices. Recently, Xie et al. fabricated flexible phosphor films by encapsulating a series of deep-trap PLMs with multicolor emissions into silica gel (Zhuang et al., 2018). The information was conveniently encoded to the film using a 405-nm laser and the decoding process was conducted by scanning with a 980-nm laser.

## Conclusions and Prospects

As promising luminescent agents, PLMs have drawn wide attentions owing to their ultra-long afterglow. In this review, we discussed the crucial breakthroughs and latest developments of different PLMs for diverse biomedical and informational applications. Inorganic PLMs with stable, potent and super-long PL were formed via effective traps and strong emitters. To overcome the low dispersibility and biocompatibility and the complexity of production, organic PLMs, consisting of purely small molecules and polymers PLMs, have been investigated.

Considering the advantages of eliminating the *in situ* excitation, PLMs exhibit enormous potential for bioimaging with super-long decay time and high SNR. Moreover, with appropriate functionalization, PLMs are ideal platforms to establish multifunctional systems in imaging-guided delivery and theranostics. Nevertheless, owing to the long reading window provided by the PL property, PLMs also possess great potential in informational technologies, such as data storage and anti-counterfeiting.

However, for the more convenient applications of PLMs, there are still several problems need to be solved. Firstly, achieving long and strong PL synchronously poses a major challenge, because strong PL requires high radioactive decay rate at the cost of a short PL. To address this problem, the radioactive decay rate needs to be moderate and the excitation transformation needs to be sufficiently efficient. However, neither of these can be realized experimentally. Secondly, for the more efficient application in bioimaging, the stable PL of inorganic or organic PLNPs in aqueous solution is essential. In addition, the color range needs to be broadened, because various visible lights are beneficial in informational applications, and efficient excitation and emission wavelengths in NIR are favorable in biomedical applications.

In conclusion, a thorough interdisciplinary understanding of chemistry, materials science, biomedicine, and information technology is required for the breakthroughs and improvements of PLMs. This emerging and promising interdisciplinary understanding of these disciplines further promotes the application of PLMs in all fields of human society.

## Author Contributions

HT, CY, and LH designed and wrote the manuscript. TW and YS provided comments and helped in finalizing the manuscript. All authors reviewed the final version of the manuscript and approved it for publication.

### Conflict of Interest Statement

The authors declare that the research was conducted in the absence of any commercial or financial relationships that could be construed as a potential conflict of interest.
